# Ambulance route optimization in a mobile ambulance dispatch system using deep neural network (DNN)

**DOI:** 10.1038/s41598-025-95048-0

**Published:** 2025-04-24

**Authors:** C. Selvan, Basha H. Anwar, Soumyalatha Naveen, Shaik Thasleem Bhanu

**Affiliations:** 1 School of Computer Science and Engineering, REVA University, Bengaluru, Karnataka India; 2 Department of Computer Science and Engineering, Rajalakshmi Institute of Technology, Chennai, Tamilnadu India; 3 Department of Computer Science and Engineering, Manipal Institute of Technology Bengaluru, Manipal Academy of Higher Education, Manipal, India; 4 Department of Electronics and Communications Engineering, Rajalakshmi Engineering College, Chennai, Tamilnadu India

**Keywords:** Computer science, Information technology

## Abstract

The ambulance dispatch system plays a crucial role in emergency medical care by ensuring efficient communication, reducing response times, and ultimately saving lives. Delays in ambulance arrival can have serious consequences for patient health and survival. To enhance emergency preparedness, decision trees are used to analyze historical data and predict ambulance demand in specific locations over time. This helps in planning the necessary number of ambulances in advance. In situations where ambulance resources are limited, a support vector machine (SVM) evaluates patient data to optimize the distribution of available ambulances, ensuring that the most critical patients receive timely medical attention. For real-time route optimization, a convolutional neural network (CNN)-based deep learning model is used to adjust ambulance routes based on current traffic and road conditions, achieving an accuracy of 99.15%. By improving dispatch efficiency and communication, the proposed machine learning-based ambulance system reduces the burden on emergency services, enhancing overall effectiveness, particularly during peak demand periods.

## Introduction

In medical situations, ambulances are frequently the initial point of contact. An effective emergency medical response can be provided by a well-maintained ambulance service system that can save lives and lessen the severity of injuries. An ambulance dispatch system dramatically aids the prompt and appropriate delivery of medical care to patients. Delaying medical care in situations where the patient is experiencing a medical emergency, such as a heart attack or stroke, can dramatically lower their chances of surviving as well. Delays in receiving medical care can make patients feel worse, which can make it more difficult to control their symptoms and lower their quality of life^1^. A good ambulance service system should ensure prompt and safe transportation of patients, which can be crucial for their recovery. For patients who need rapid medical attention, ambulance services are essential for providing both transportation and medical aid^2^. A delayed reaction can greatly impact a patient’s chances of survival^3^, and in rare circumstances, it can even result in death. The Bombay government has responded to this occurrence by enhancing ambulance services, including expanding the number of ambulances and strategically placing them according to demand and traffic patterns. This emphasizes the need for effective dispatching systems for ambulances and real-time tracking systems to guarantee that they can reach patients in need as soon as feasible.

The ambulance service offers rapid medical care, which in many cases can lead to differences between life and death. It is impossible to exaggerate the value of ambulance services in an emergency. The ambulance service can offer prompt medical attention in emergencies where time is essential, such as a heart attack or stroke, which can save lives. Furthermore, essential to ensuring that patients receive the required medical care is transporting patients to hospitals, clinics, or other healthcare facilities via ambulance services^4^. Ambulance services are frequently the sole way for residents of these communities to access medical care and transportation. In addition to providing medical treatment and transportation during natural disasters such as earthquakes, floods, and hurricanes, ambulance services are essential to disaster management. Whether they live in rural or urban locations, end users could benefit from prompt administration of patient medication without any delays by using the ambulance application system.

The contribution of this proposed work is as follows:

- Utilizes a Deep Neural Network (DNN) to dynamically optimize ambulance routes based on real-time traffic and road conditions, reducing response times.
- Implements machine learning techniques to forecast ambulance demand, ensuring efficient deployment and preparedness in high-risk areas.
- Uses Support Vector Machines (SVM) to prioritize ambulance allocation, ensuring critically ill patients receive timely medical attention.
- Enhances communication and dispatch efficiency, minimizing delays and optimizing resource utilization during peak demand periods.

## Literature survey

### Ambulance and medical services

Systems for dispatching ambulances have the potential to greatly increase emergency medical services. A thorough analysis of ambulance location and dispatching tactics is given by Lim et al. Response times are impacted by the geography of the region where the ambulance service is located. Ambulance response times can also be slowed by hilly terrain, dense forest cover, and other natural obstacles^5^. In their research, H.Setzler et al. assessed the response times of ambulances in urban and rural locations and examined how variables such as distance, traffic conditions, and population density affect response times. Owing to the distance between population canters, reaction times in rural areas with sparse populations may be slower^6^.The authors examine how call volume, population density, and ambulance accessibility affect response times. Each policy for ambulance service has advantages and disadvantages, and the efficiency of a policy depends on the particulars of the emergency medical services (EMS) system. Similarly, alternative transportation policies might work well in rural areas but be too expensive to use in cities^7^.

### Virtual routing problem

Nanwani et al. developed an ambulance monitoring system^8^. It employs GPS and GSM technology to track the location of the ambulance and sends real-time updates to the hospital and the patient’s family. A system that employs IoT technology to identify accidents and promptly calls for an ambulance was later proposed by Karmokar et al. The system also has a GPS tracking feature to aid the ambulance in swiftly locating the scene of the accident. This information can be used to evaluate the effectiveness of EMS services and pinpoint areas for development^9^. To estimate the demand for ambulance services in various places and accelerate response times, W. Zhang et al. launched an emergency ambulance dispatching system via machine learning techniques^10^. Hajiali proposed a real-time ambulance dispatching system that prioritizes emergency cases and uses route optimization techniques to speed up response times. This system gives drivers, medical facilities, and other stakeholders access to real-time updates, which enhances communication and coordination between all parties and significantly improves the performance of the ambulance service and patient outcomes^11^. Despite extensive research on VRP issues, some real-world VRP applications remain extremely difficult to solve^12^. The traveling salesman problem (TSP), one of the most actively researched combinatorial optimization problems, was first proposed by Menger and is connected to the VRP^13^. TSP seeks to find the shortest circular path to travel through all customers without considering several practical constraints, such as capacity and time^14^. There is no reliable ML-assisted VRP research platform for training and testing. The research communities must collaborate to develop an integrated platform with libraries and tools for both ML and optimization, which is the focus of^15^.

The key research gaps identified in this literature review are as follows:

- There is a lack of models that can adapt in realtime without requiring frequent retraining or recalibration. Current models such as decision trees or static optimization algorithms do not handle dynamic, time-dependent factors such as traffic congestion or accidents effectively.
- Most models optimize only the initial route, but real-time dynamic rerouting algorithms that adjust routes on the basis of ongoing changes, such as live traffic data, roadblocks, or emergency reprioritization, are needed.
- Current optimization techniques often fail to scale effectively when applied to large metropolitan areas.There is a need for models that handle the computational complexity of optimizing ambulance routes across large geographic areas with high-dimensional input data (e.g., real-time traffic data, multiple emergencies, different vehicle types).

## Proposed methodology

**Fig. 1:**
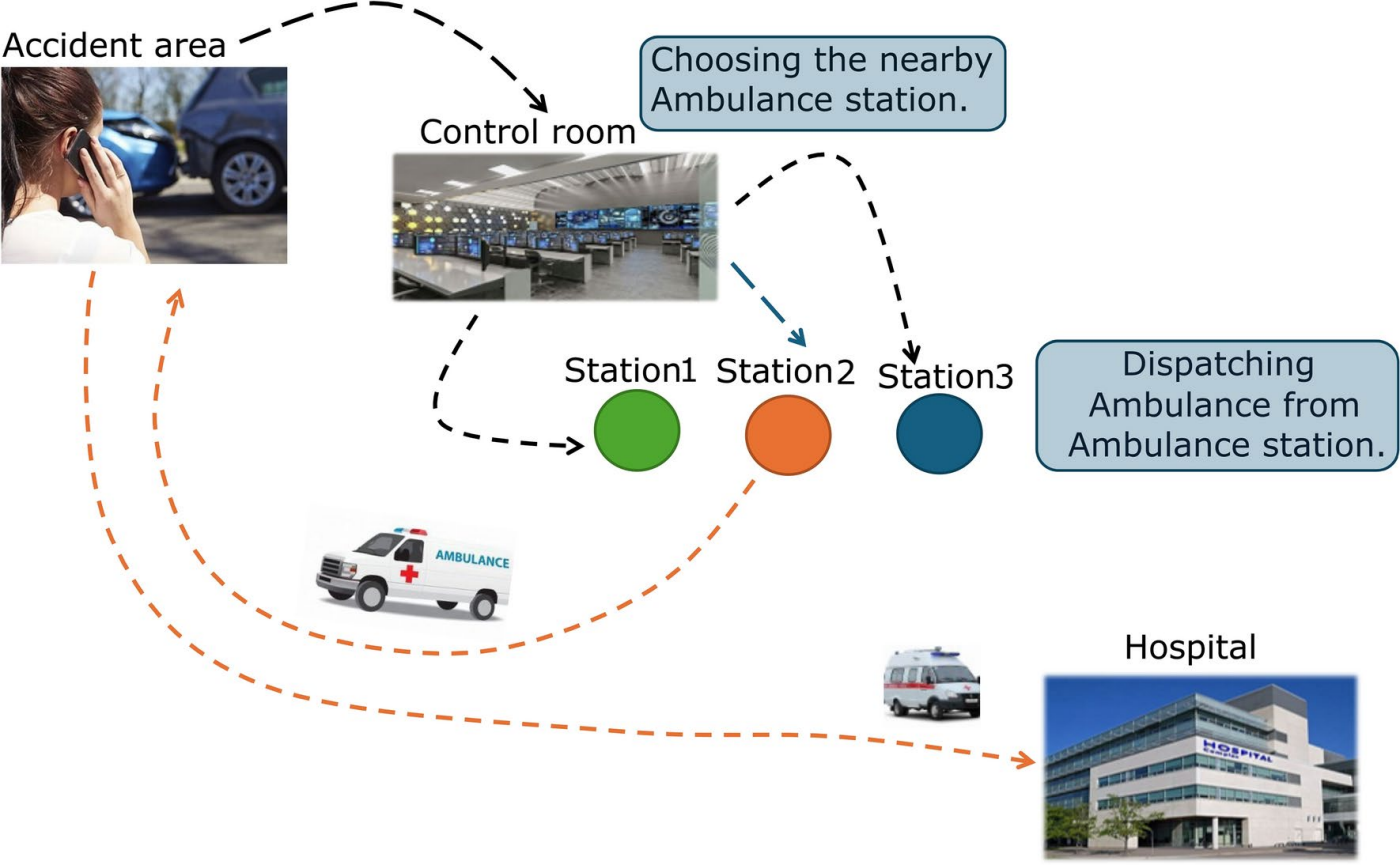
Depicts a typical ambulance dispatch scenario.

The proposed system is divided into three phases. First, the dataset is collected and analyzed via a decision tree algorithm, which predicts the need for ambulances on the basis of collected historical data, allowing ambulances to be available around the clock day^16^. In the second phase, the data are sent for model training, which is then directed to the SVM algorithm with GPS technology. Figure 1 shows the overall scenario of ambulance dispatch to the end users.

**Fig. 2:**
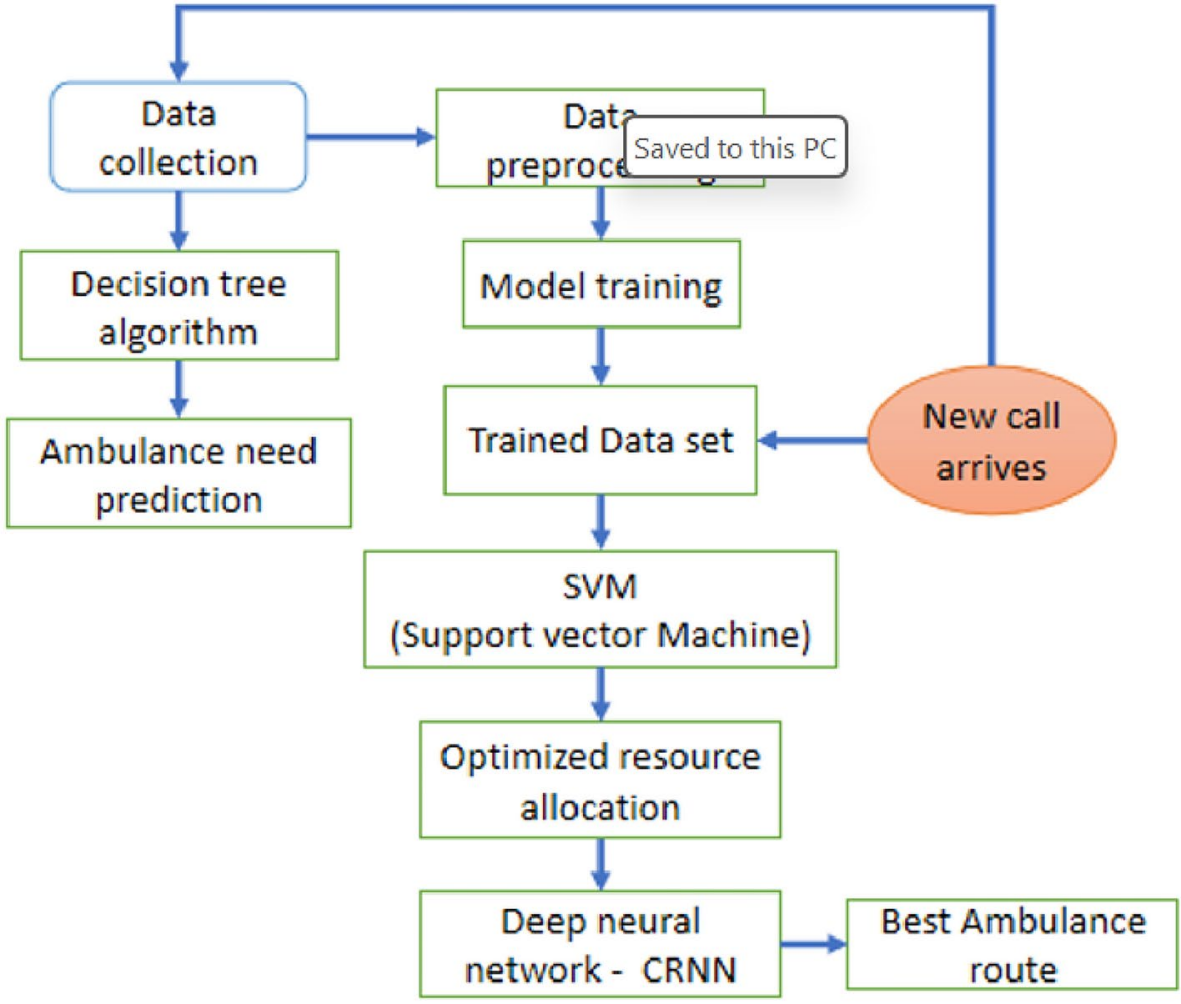
Ambulance dispatch system architecture.

Figure 2 shows each phase’s direction flow, clearly stating each phase input and output that is delivered. Finally, to achieve an optimized ambulance dispatch that provides the shortest path to the hospital,a CRNN is used, which provides a quick response to dispatch the ambulance services. Traditional methods such as linear programming (LP) and integer programming (IP) are well-suited for deterministic and linear problems. However, ambulance route optimization is a highly dynamic and nonlinear problem involving multiple real-time factors, such as traffic conditions, road closures, and patient priorities. DNNs excel in learning complex patterns from historical and real-time data, making them particularly effective in dynamic routing scenarios. Unlike LP and GA, which require explicit modeling of constraints and objective functions, DNNs can autonomously learn these relationships from large datasets.

Pre-processing is a crucial step in data analysis that enhances the quality and reliability of input data before model training. It involves data cleaning, normalization, and transformation to handle missing values, remove inconsistencies, and standardize formats. Feature selection and extraction help in reducing dimensionality, improving model efficiency. In ambulance route optimization, pre-processing includes filtering real-time traffic data, encoding categorical variables, and normalizing GPS coordinates for accurate predictions. Proper pre-processing ensures that the model learns meaningful patterns, minimizes noise, and improves overall performance, leading to more accurate and efficient ambulance dispatch decisions in emergency scenarios.

### Ambulance needs prediction

Emergency services can be more ready and responsive if they anticipate when an ambulance is needed. This is crucial in circumstances such as medical emergencies or serious injuries, where every second counts^17^. The basic assumption for the Mobile Ambulance Dispatch System is the availability of the ambulance, traffic conditions, priority for the patients, ambulance capacity and the present location of the ambulance.Decision trees can be effective tools when determining whether an ambulance is required before an accident. A decision tree is a hierarchical model that represents decisions and their potential outcomes. The tree nodes represent decision points, and the edges represent potential outcomes of those decisions^18^. After being built and trained with historical data, the decision tree can be used to predict^19^the demand for an ambulance in realtime. When a new emergency call arrives, the system can feed the relevant data into the decision tree and use it to forecast the likelihood of requiring an ambulance. An ambulance can be dispatched if the likelihood is high^20^. Let X represent all decisions and Y represent all outcomes. The following equations can be used to represent the decision tree:

- For each decision i $\in$ X, let $A_{i}$ be a binary variable that takes a value of 1 if the decision is made and 0 otherwise. Then, we have
  1 $$\begin{aligned} \Sigma _{i}^{X} A_{i}=1 \end{aligned}$$
  This equation ensures that only one decision is made at each decision point.
- For each outcome m $\in$ C, let $Y_{m}$ be a binary variable that takes the value 1 if the outcome occurs and 0 otherwise. Then we have:
  2 $$\begin{aligned} Y_{m}=\Pi _{i\in DL}m(n)^{X_{n}}*\Pi _{i\in D}(1-m(n))^{(1-p_{n})} \end{aligned}$$
- The expected value of the decision tree can be calculated using the following equation:
  3 $$\begin{aligned} R=\Sigma _{j \in Y}Q_{j} X C_{j} \end{aligned}$$
  where $Q_j$ is the value associated with outcome j. This equation calculates the expected value of the tree by summing the value of each outcome weighted by its probability.

### Support vector machine

Support vector machines (SVMs) are a type of supervised machine learning algorithm that can be used for classification and regression analysis and highly helps in prioritizing ambulance services on the basis of the severity of the patient’s condition. The SVM assumes that there exists a hyperplane (or decision boundary) separating feasible route choices on the basis of input factors such as traffic, distance, and case priority. The mathematical representation of SVM for a binary classification problem can be described as follows: SVM aims to find a hyperplane that separates the input vectors into two classes with the maximum margin between them.

The equation can represent the hyperplane:

4 $$\begin{aligned} L+g=0 \end{aligned}$$

where L is a vector normal to the hyperplane and g is the bias term that shifts the hyperplane. The decision function for the SVM is defined as,

5 $$\begin{aligned} f(L)=sign(O. L+g) \end{aligned}$$

where sign is the sign function that returns $-$1 or 1 depending on the polarity of the input value.

The margin is defined as the distance between the hyperplane and the closest data points of each class. The distance between a point Ln and the hyperplane is computed as:

6 $$\begin{aligned} distance(Ln,hyperplane)=|O. Ln+g|/||O|| \end{aligned}$$

where ||*O*|| represents the Euclidean norm of O. The optimization problem for the SVM can be formulated as

7 $$\begin{aligned} minimize\left( \frac{1}{2}\right) ||O||^2 \text {subject to} M_{n}(O. Ln +g)>=1, n=1,..e \end{aligned}$$

where the first term in the objective function represents the margin and the second term represents the constraint that all the data points are correctly classified with a margin of at least 1. This is known as the primal problem of SVM^21^. The dual problem is subject to

8 $$\begin{aligned} \Sigma \alpha nMn=0, 0<=\alpha n <=T, n=1,...e \end{aligned}$$

where $\alpha n$ are the Lagrange multipliers, and T is a hyperparameter that controls the trade-off between maximizing the margin and minimizing the classification error. The decision function for SVM can be computed as:

9 $$\begin{aligned} f(L)=sign(\Sigma (\alpha nMnLn. L+g)) \end{aligned}$$

where the sum is taken over all support vectors, which are the data points that lie on the margin or violate the margin constraint.

### CRNN - Anticipating the best route

**Fig. 3:**
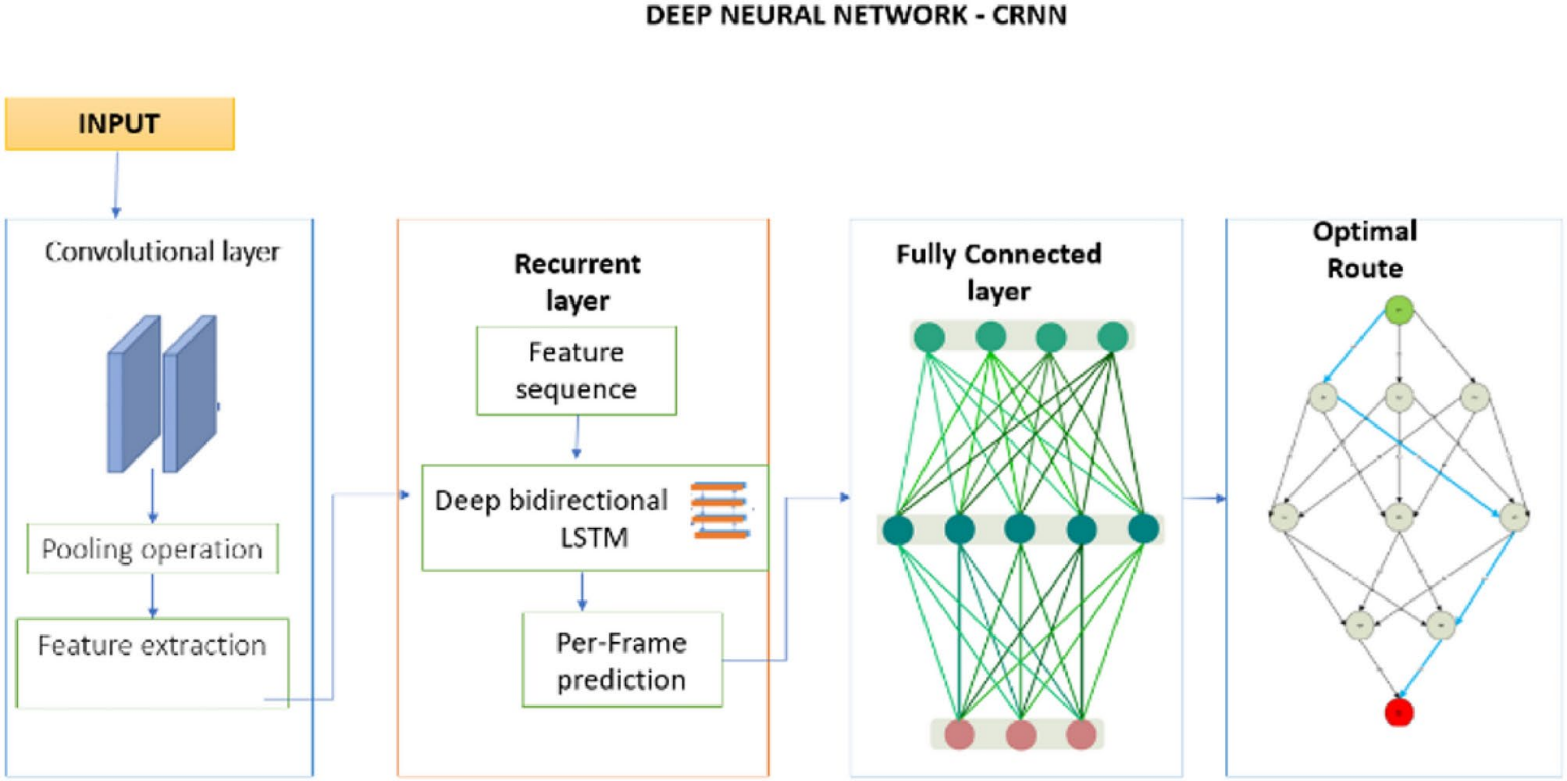
Working of CRNN in finding the best route for the ambulance.

A CRNN can predict the best path for an ambulance traveling a short distance. The network’s input could be a set of GPS coordinates or other location information, and its output could be the anticipated path the ambulance will take. In an ambulance dispatch system, a convolutional recurrent neural network (CRNN) can be used to combine the strengths of both convolutional and recurrent neural networks to predict the best path for an ambulance^22^. When these features are used to create a map of the area, potential routes for the ambulance to take to reach the patient as quickly and efficiently as possible can be identified^23^. The CRNN can make route adjustments on the fly, continuously optimizing the path as new information arrives. The output of the CRNN model is the continuous update on the optimal route for the ambulance to reach the emergency location. The working of the CRNN in finding the best route for the ambulance is depicted in Fig. 3.

During the prediction process, the CRNN considers the patient’s location, the ambulance’s current location, and other relevant information, such as real-time traffic updates, and then generates a series of hidden states that represent the network’s evolving understanding of the context^24^. When a new emergency call arrives, the pertinent information is entered into the trained DNN to receive a suggested ambulance route^25,26^.

### Workflow sequence of CRNN

#### Input given

TThe input sequence contains the ambulance’s present location, destination location, and other important information.

#### Convolutional layer

To extract features from the sequence, the input data are processed and filtered multiple times through n convolutional layers. The output of the k-th convolutional layer can be represented as:

10 $$\begin{aligned} l_m= Conv(C_M * l_{m-1} + k_m) \end{aligned}$$

where $l_{m-1}$ denotes the output of the (m-1)-th convolutional layer, $C_M$ and $k_m$ are the weights and biases of the m-th convolutional layer, and * denotes the convolution operation.

#### Recurrent layer

To capture the temporal dependencies in the sequence, a recurrent layer is applied after the feature mappings from the convolutional layer. The output of the m-th recurrent layer can be represented as:

11 $$\begin{aligned} l_m = RNN(l_{m-1},y) \end{aligned}$$

where $l_{m-1}$ denotes the output of the (m-1)-th recurrent layer, y denotes the input sequence and RNN is a recurrent neural network cell(LSTM)

#### Fully connected layer

The optimum path for the ambulance to take to get to the destination is predicted using the hidden states from the recurrent layer 3. The output of the m-th fully connected layer can be represented as:

12 $$\begin{aligned} l_m = f(C_M * l_{m-1} + k_m) \end{aligned}$$

where $l_{m-1}$ denotes the output of the (m-1)-th fully connected layer, $C_M$ and $k_m$ are the weights and biases of the m-th fully connected layer, and f is a non-linear activation function.

#### Training and prediction

Backpropagation and gradient descent are used to train the CRNN to reduce the difference between the expected and actual outputs. As a result, the CRNN algorithm is better able to anticipate the optimum path for an ambulance to travel quickly since it can consider real-time updates of traffic conditions, road closures, and other pertinent data.

## Result analysis

The incorporation of machine learning algorithms into ambulance dispatch systems has several advantages, such as faster emergency response times, lower mortality rates, and higher-quality emergency care overall. Table. 1 shows sample data taken into account for experimental analysis of the proposed system. The Kaggle dataset^27^on ambulance route selection, which is composed of 605 files of road conditions and the time duration to reach the emergency point, is employed. Among the 605 files, 80% of the files were used for training the deep learning model, whereas 20% of the data were used for testing. The generalizability of the proposed model is an important consideration, particularly for varying geographic regions and datasets. While the model was trained and validated using real-world ambulance dispatch data, differences in road infrastructure, traffic patterns, and urban planning may impact its performance in other regions. Grid search was utilized for hyperparameter tuning, evaluating multiple configurations to optimize model performance. Various learning rates (0.0001, 0.001, 0.01), hidden layers (3, 5, 7), neurons per layer (32, 64, 128), batch sizes (16, 32, 64), activation functions (ReLU, Tanh), and optimizers (Adam, RMSprop) were tested. The best hyperparameters were selected based on Mean Absolute Error (MAE) and Mean Squared Error (MSE) to ensure optimal generalization to unseen data.

**Table 1:** Sample dataset of patient emergency calls.

S. no	Sex	Age	Arrival mode	Injury	Complaint	Mental	Pain	Call time	Call received location	Ambulance no.
1	2	59	2	1	Acute epigastric pain	1	1	20:41	Kangan	A4
2	1	56	2	1	Pain, leg	1	1	06:03	Srinagar	A7
3	2	58	2	1	Epigastric pain	1	1	09:07	Samboora	A2
4	1	63	2	1	Fracture	2	1	15:32	Baramulla	A1
5	2	59	2	1	Pain	1	3	01:50	Srinagar	A6
6	2	52	1	1	Headache	1	1	13:08	Samboora	A2
7	1	64	1	1	Gingival swelling	1	1	19:25	Srinagar	A9
8	2	61	2	2	Chin pain	1	1	04:16	Waniyar	A11
9	1	49	1	1	RUQ pain	1	3	09:52	Hig Colony	A15
10	1	56	2	2	Open Wound	1	1	16:41	Rath Pora	A12
11	2	51	2	1	Skin rash	1	1	23:15	Chhatbal	A13

A decision tree is used for analyzing large amounts of data on patient outcomes, emergency response times. Table 2 shows the data fields taken into account for predicting the areas where emergency medical care services are needed. Performance metrics were selected to align with real-world ambulance dispatch system requirements, focusing on both accuracy and efficiency. Mean Absolute Error (MAE) and Mean Squared Error (MSE) were used to evaluate route prediction accuracy, ensuring minimal deviation from optimal routes. Computational time was considered crucial, as rapid decision-making is essential in emergency response. Lower error rates contribute to precise route optimization, reducing response times, while efficient computation ensures real-time applicability. These metrics collectively reflect the system’s ability to provide fast and accurate ambulance routing, directly impacting emergency medical service efficiency.

**Table 2:** Extracted data for decision tree analysis.

Predictor variables for decision tree analysis	Need for dataset inclusion
Day of a week	To account for the ambulances nonstationary nature
Hour in a day	To account for the ambulances nonstationary nature
Number of calls received in a day	To account for the effect of call volumes
Clear rate of an ambulance at Emergency Department per hour	To account for the effect of call volumes
Number of Ambulances Currently in Emergency Department	To account for the current status of Ambulance

**Fig. 4:**
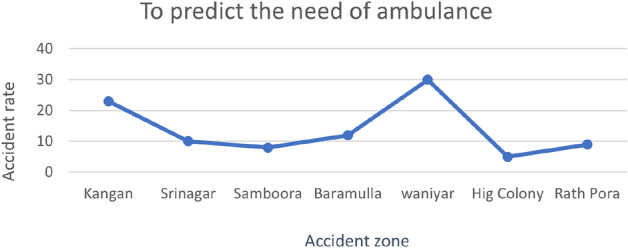
Graph representing higher emergency medical care needed areas.

Figure 4 shows a graph that clearly shows the area where many emergency calls were received and,on the basis of the ambulance dispatch system, is trained.

**Fig. 5:**
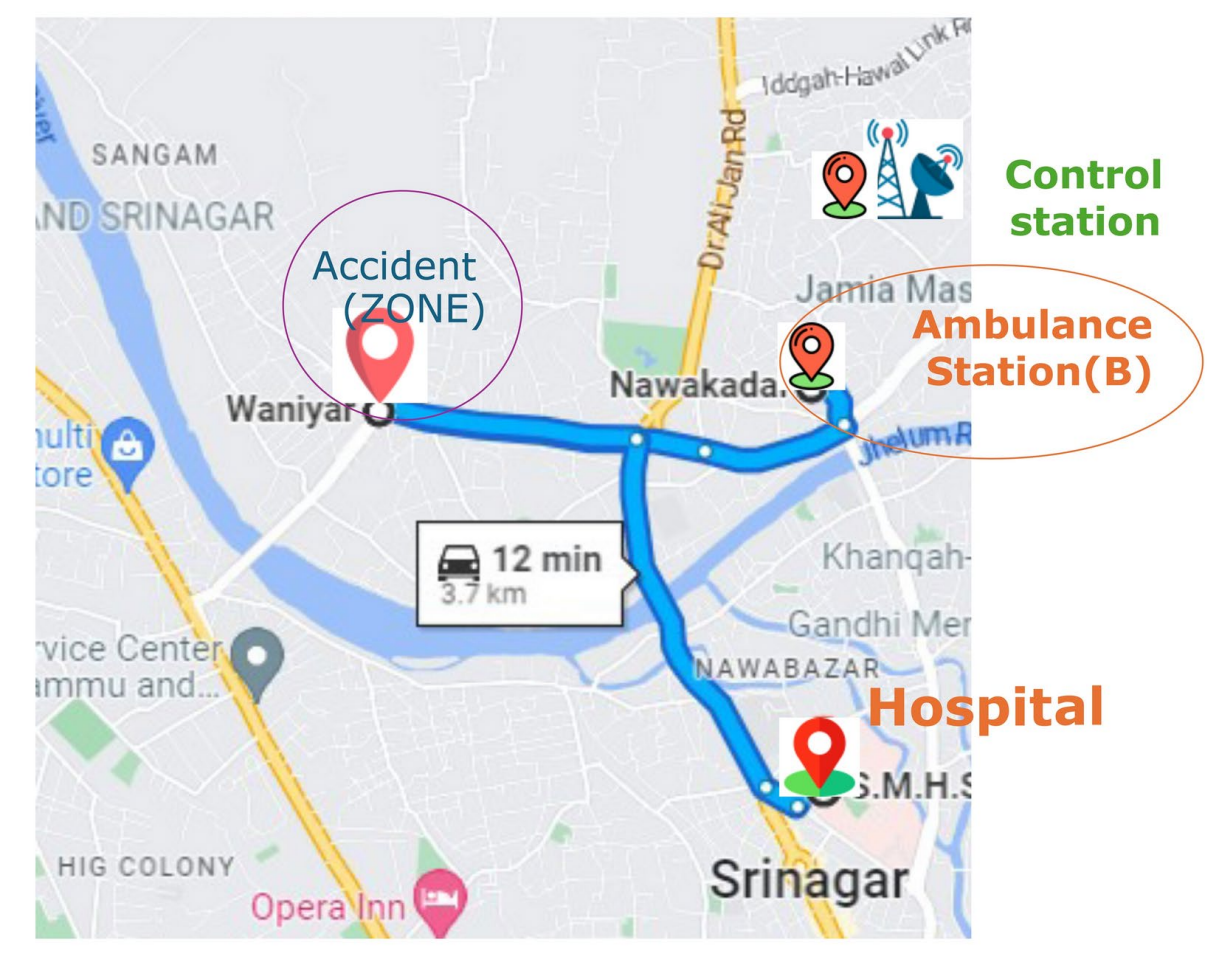
Distance from Ambulance station B to hospital.

**Fig. 6:**
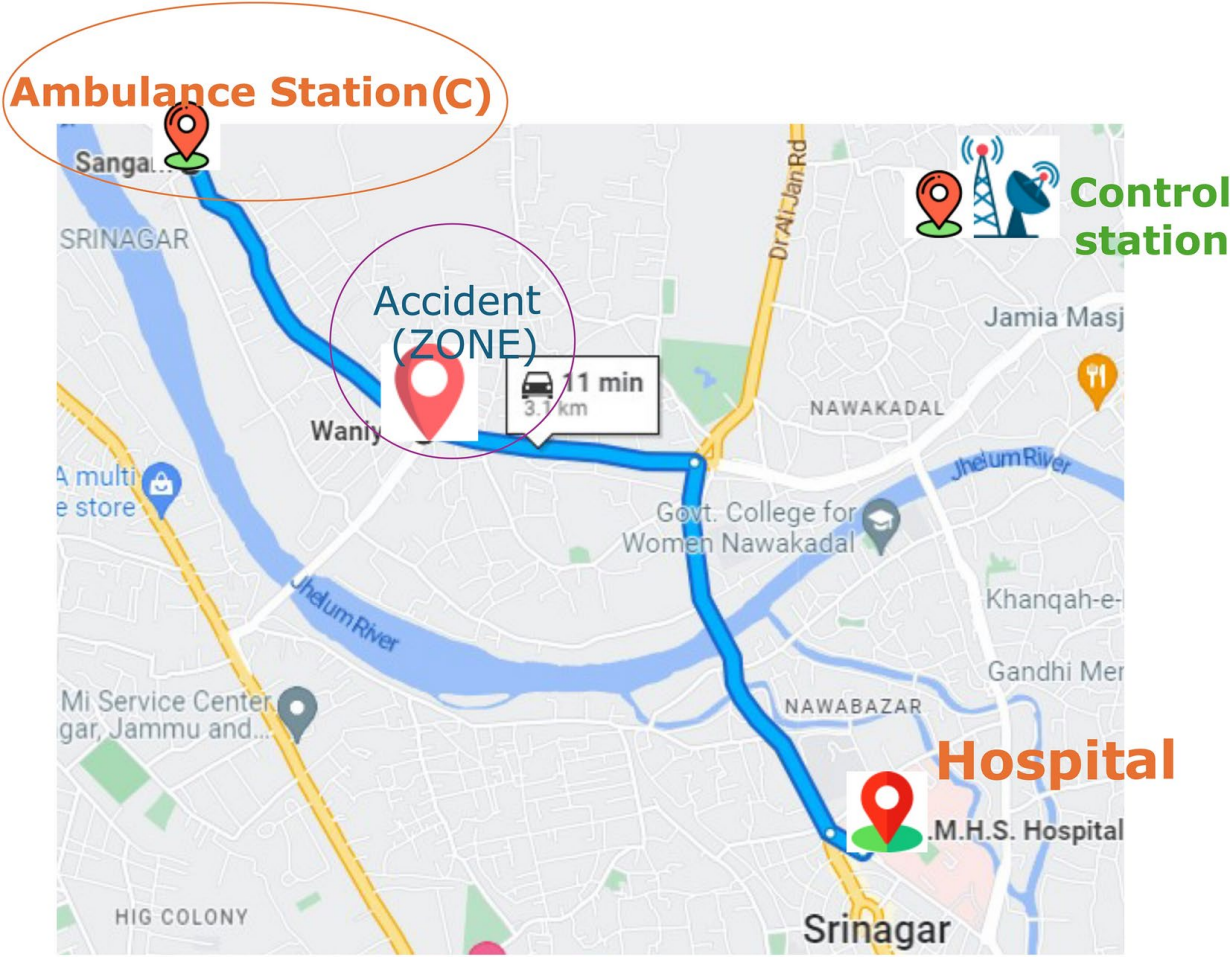
Distance from Ambulance station C to hospital.

This can help dispatchers prioritize calls more accurately and effectively with limited ambulance resources.The distance from Ambulance Station B and C to the hospital is depicted in Figs. 5 and 6 respectively.

**Fig. 7:**
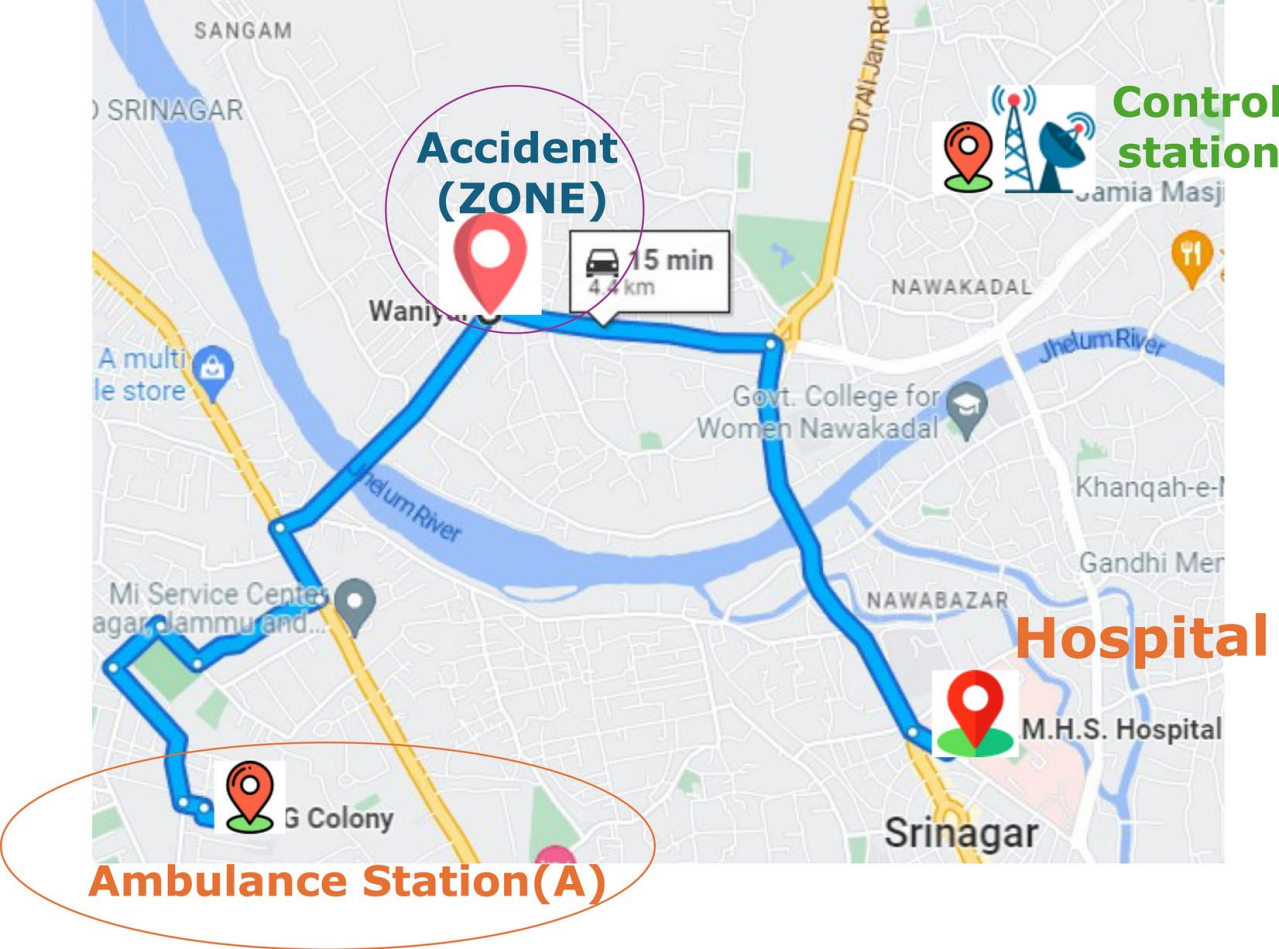
Distance from Ambulance station A to hospital.

**Fig. 8:**
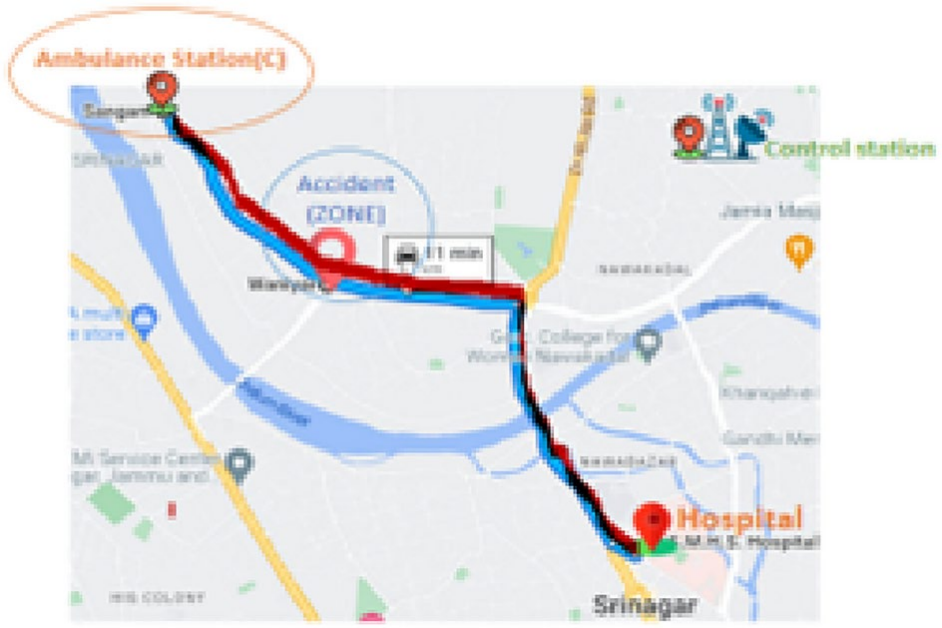
Depicting the optimal path through CRNN algorithm.

After the SVM optimized the ambulance distribution to stations under the predicted need of ambulances computed by the decision tree algorithm on the basis of the emergency call history, the CRNN shifted this proposed idea to a more effective part of implementing ambulance services to the public as fast as possible, as this is a lifesaving minute in many people’s lives.The distance from Ambulance Station A to the hospital and Depicting the optimal path through the CRNN algorithm is presented in Figs. 7 and 8 respectively.

**Fig. 9:**
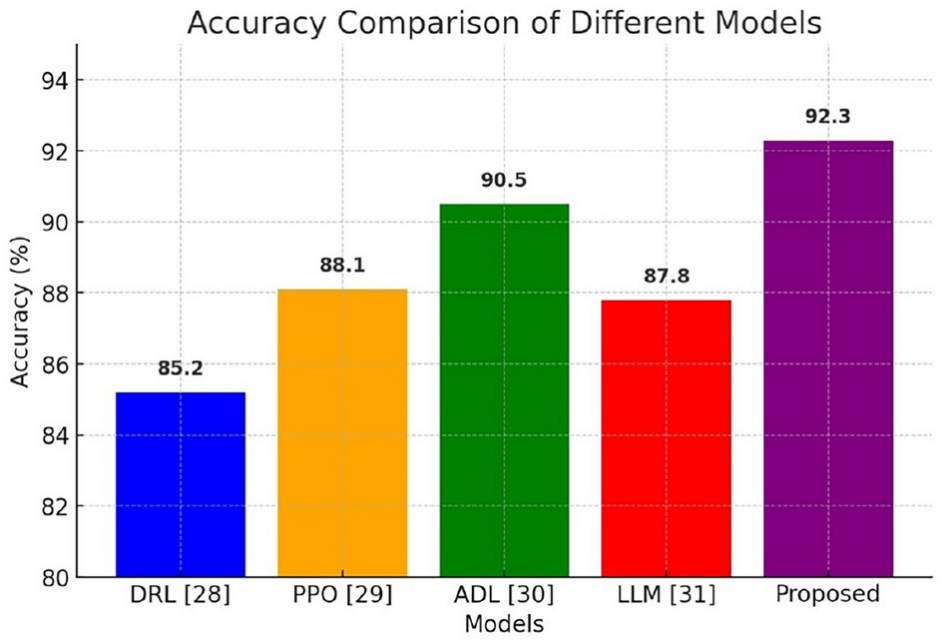
Efficiency of machine learning algorithms in routing.

In this sample scenario, an experiment is conducted, and a call is received from an accident zone to a nearby control station for emergency medical support. Our goal is not only to predict the shortest path but also to determine an optimized route without any major disturbances in the system. This is an important factor for finding the optimized path via the CRNN. In addition to these input numbers of ambulances at each ambulance station, road traffic is processed through the CRNN layers, and the final prediction is performed. Figure 9 shows an illustration of some of the machine learning algorithms involved in vehicle routing.

Various machinelearning algorithms take part in providing the best route to solve for vehicles to provide faster responses to the public. The proposed method implements its optimized ambulance via the CRNN algorithm.

Computational time in ambulance route optimization denotes the duration required for the model to ingest input data and formulate an optimal routing solution. In emergency medical services, expeditious decision-making is paramount, as even marginal delays can significantly affect patient survival rates. Computational efficiency is influenced by algorithmic complexity, dataset scale, and real-time processing constraints. Conventional optimization techniques often incur prolonged runtimes due to iterative computations, whereas deep learning models, once trained, facilitate near-instantaneous predictions. Minimizing computational time is crucial for real-time implementation, enabling ambulances to swiftly adapt to evolving traffic dynamics and enhance emergency response efficacy. The computational time is compared with the state of the art methodologies and is presented in Table 3.

**Table 3:** Performance comparison with state of the art methods.

Parameters	DRL^28^	PPO^29^	ADL^30^	LLM^31^	Proposed
Accuracy	85.2	88.1	90.5	87.8	92.3
Precision	92.4	85.9	89.2	86.5	91.0
Recall	80.1	84.5	88.7	85.2	90.8
F1 score	81.2	85.2	89.0	85.8	90.8
Computational time	1200 ms	980 ms	850 ms	1100 ms	720 ms

**Fig. 10:**
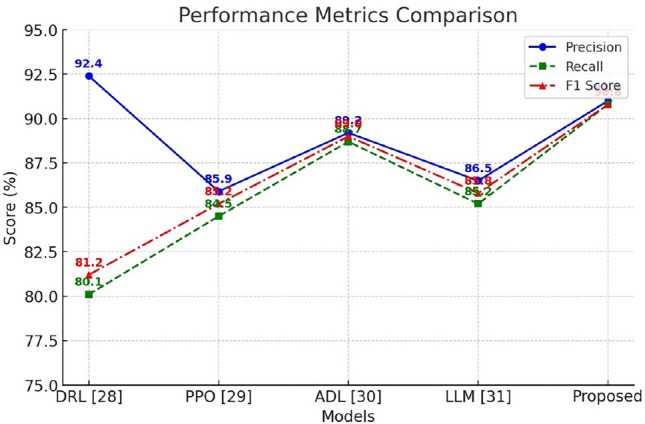
Comparison of Performance with State of art methodologies.

The Fig. 10 illustrates the performance metrics-Precision, Recall, and F1 Score-across different models, including DRL, PPO, ADL, LLM, and the Proposed model. Precision reflects the proportion of correctly predicted positive cases, while Recall indicates the model’s ability to capture all relevant cases. F1 Score balances these two metrics, offering a comprehensive measure of model effectiveness. The Proposed model outperforms others, achieving high Precision (91.0%), Recall (90.8%), and F1 Score (90.8%), indicating improved accuracy and reliability in ambulance route optimization. ADL also performs well, whereas PPO and LLM show moderate performance. DRL demonstrates high Precision but lower Recall, suggesting potential trade-offs in prediction consistency. The visualization highlights the effectiveness of the Proposed model in optimizing emergency response routing.

**Fig. 11:**
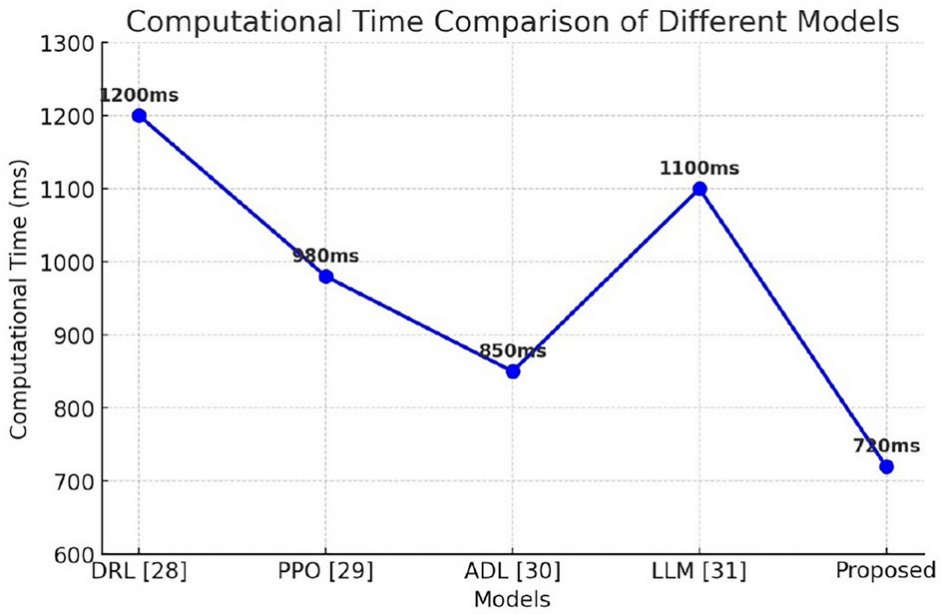
Comparison of Computational Time with State of art methodologies.

Figure 11 illustrates the computational time of different models used in ambulance route optimization. Among the models compared, DRL exhibits the highest computational time (1200ms), indicating slower processing, while PPO (980ms) and LLM (1100ms) show moderate efficiency. ADL demonstrates improved performance with a reduced computational time of 850ms. The proposed model achieves the lowest computational time (720ms), highlighting its superior efficiency in real-time applications. This reduction in computational time is crucial for emergency medical services, as faster processing enables rapid decision-making, ultimately leading to improved ambulance dispatch and response times in critical situations.

As shown in Table 4, Sensitivity analysis is conducted to assess the robustness of the proposed model by evaluating its performance under varying real-world conditions. Key factors such as traffic congestion, road closures, weather conditions, and emergency request density are systematically altered to determine their impact on model predictions. This involves simulating different traffic levels, introducing sudden road diversions, and testing the model under fluctuating ambulance demand. By analyzing changes in accuracy, computational time, and route efficiency, the study ensures that the model remains reliable across diverse scenarios. These insights strengthen the practical applicability of the model, enhancing its adaptability for real-world ambulance dispatch systems.

**Table 4:** Sensitivity analysis under various conditions.

Scenario	Traffic density	Road closures	Emergency requests	Accuracy (%)	Computational time (ms)	Route efficiency (%)
Baseline (Normal)	Moderate	None	Standard	92.3	720	89.5
High traffic	High	None	Standard	90.1	850	85.2
Low traffic	Low	None	Standard	94.5	680	92.1
Road closures	Moderate	Multiple	Standard	88.7	940	80.9
High emergency	Moderate	None	High	89.8	810	83.5

## Conclusion

Delayed arrival at an ambulance can result in a delay in medical attention, which can cause the patient’s condition to worsen, leading to long-term health complications. The proposed ambulance dispatch system enables effective communication between emergency services, medical professionals, and the public. The proposed ambulance service system ensures that the right number of ambulances is readily available when needed, which is computed with the help of a decision tree algorithm. This can be particularly important in rural or remote areas where medical facilities are limited. SVM is a powerful tool that can be used to prioritize ambulance services on the basis of the severity of the patient’s condition. This would enable the system to improve its performance over time without requiring complete model retraining, especially when exposed to new traffic patterns or emergency distribution trends. The Kaggle dataset, while valuable for training and evaluating the model, may have limitations in terms of representativeness and potential biases. It may not fully capture real-world variations in traffic patterns, emergency request distributions, or regional infrastructure differences. Additionally, biases in data collection, such as underrepresentation of rural areas or specific emergency scenarios, could impact model generalizability. These factors may affect the reliability of predictions when applied to different geographic regions. To mitigate these limitations, future work will incorporate diverse real-world datasets and domain adaptation techniques to enhance the model’s robustness and applicability.

## Data Availability

Publically available dataset is used and available in Kaggle [Ambulance Dataset], https://www.kaggle.com/datasets/tabarkarajab/ambulance-dataset.
